# Supplementary material for: Content-rich biological network constructed by mining PubMed abstracts
Source: BMC Bioinformatics. 2004 Oct 8;5:147. doi: 10.1186/1471-2105-5-147 (PMC528731; doi:10.1186/1471-2105-5-147)
Supplement: Additional File 5 — The original Chilibot query results of the term "long-term potentiation (LTP)" and 22 other terms, limiting the latest references analyzed to the years 1990, 1995, 2000, and 2004. [file 1471-2105-5-147-S5.bz2 › chilibotAdditionalFile5/ltp2000/html/AMPA_SYNAPTOTAGMIN.html]

 


 **AMPA** and **SYNAPTOTAGMIN** 
  
Found 2 abstracts in PubMed,  **2 abstracts were retrieved and analyzed**.  


---

 Search Google  |
 PDF files only 
|  EDU domain only 

---

- Mol Cell Neurosci, 2000   **Temporal appearance of the presynaptic cytomatrix protein bassoon during synaptogenesis.** .
  Bassoon is a 420 kDa presynaptic cytomatrix protein potentially involved in the structural organization of neurotransmitter release sites.
  In this study, we have investigated a possible role for Bassoon in synaptogenesis and in defining synaptic vesicle recycling sites.
  We find that it is expressed at early stages of neuronal differentiation in which it is selectively sorted into axons.
  As synaptogenesis begins, Bassoon clusters appear along dendritic profiles simultaneously with **synaptotagmin** I, sites of synaptic vesicle recycling, and the acquisition of functional excitatory and inhibitory synapses.
  A role for Bassoon in the assembly of excitatory and inhibitory synapses is supported by the colocalization of Bassoon clusters with clusters of GKAP and **AMPA** receptors as well as GABA A receptors.
  These data indicate that the recruitment of Bassoon is an early step in the formation of synaptic junctions.
